# The Effect of Zonisamide and Ethanol on Various Types of Memory in Rats

**DOI:** 10.3390/ijerph20031815

**Published:** 2023-01-18

**Authors:** Bogusława Pietrzak, Agata Krupa-Burtnik, Ewa Zwierzyńska

**Affiliations:** Department of Pharmacodynamics, Medical University of Lodz, Muszyńskiego 1, 90-151 Łódź, Poland

**Keywords:** addictive behaviors, ethanol, memory, rats, zonisamide

## Abstract

*Background:* Antiepileptic drugs might be useful in the treatment of alcohol use disorder. One of these drugs is zonisamide, which has been found to decrease alcohol intake and cravings. An important structure in the pathophysiology of addiction is the hippocampus. Memory deficits, which frequently occur in alcoholics, are associated with ethanol-induced changes in hippocampal plasticity and neurogenesis. The aim of this study was to assess the potential protective effect of zonisamide on memory in rats receiving alcohol and after the discontinuation of its administration. *Methods:* Wistar rats (n = 43) were tested in four behavioral models, namely: Morris water maze (MWM), passive avoidance (PA), contextual fear conditioning (CFC), and cued fear conditioning (CuFC). *Results:* Zonisamide co-administered with ethanol impaired spatial memory in MWM, but the drug did not affect memory in PA. However, the beneficial effect of zonisamide was observed after the discontinuation of ethanol administration, which was associated with the improvement of associative memory in CFC and the alleviation of alcohol-induced locomotor disturbances in CuFC. *Conclusion:* Zonisamide has a differential influence on memory, which depends inter alia on type of the memory, length of ethanol administration, or its absence.

## 1. Introduction

Anticonvulsant drugs could possibly be used in the treatment of alcohol use disorder. Both first-generation and newer antiepileptics have shown some efficacy in reducing alcohol intake in clinical trials [1,2,3]. Zonisamide is a new-generation anticonvulsant with a similar mechanism of action to topiramate. The drug blocks sodium channels [4] and calcium channels [5], reduces glutamate transmission, and enhances GABA(-ergic transmission [6]. Zonisamide is also a weak inhibitor of carbonic anhydrase, but this effect does not seem to be clinically relevant [7].

It is known that some antiepileptic drugs may impair cognition. One example is topiramate, which is more likely to elicit cognitive deficits, and affects multiple domains including memory, language, and attention [8]. It has been reported that zonisamide may also cause mild to moderate cognitive adverse effects in epilepsy patients [9]. However, similar to topiramate, zonisamide may also reduce alcohol consumption and cravings, as shown in both preclinical [10,11] and clinical studies [2,12,13]. A comparative clinical study showed that zonisamide has similar effects to topiramate in reducing drinking, including amount of the drinks consumed per day, percent days drinking, and percent days heavy drinking. While both drugs might produce some modest cognitive deficits, those associated with zonisamide are milder [2]. Furthermore, zonisamide seems to be more effective than diazepam when treating alcohol withdrawal syndrome [14].

It is known that hippocampal dysregulation plays an important role in the pathophysiology of alcohol use disorder. Learning and memory deficits are directly influenced by chronic alcohol consumption, as long-term alcohol use may provoke cognitive dysfunction and memory problems associated with an adverse impact on hippocampal plasticity, inhibition of hippocampal neurogenesis, and changes in the hippocampal structure [15,16]. Being a drug with a multidirectional mechanism of action, zonisamide could modulate the activity of the hippocampus and neurotransmitter pathways involved in memory processes. Our previous pharmaco-electroencephalography study found that zonisamide decreased alcohol- and abstinence-induced changes in selected brain structures involving the hippocampus [17]. Hence, the aim of the present study was to assess the effect of zonisamide on memory processes during prolonged exposure to ethanol and also after its discontinuation. The study employs various behavioral models: Morris water maze (spatial memory), passive avoidance (memory associated with the fear), cued fear conditioning, and contextual fear conditioning (memory associated with the fear).

## 2. Materials and Methods

### 2.1. Animals

The study used 43 male Wistar rats weighing 240–280 g. The animals were obtained from the Medical University of Lodz, Poland, and housed under normal laboratory conditions (20–22 °C, 12 h light/12 h of dark cycle) with access to commercial chow. Experiments were performed between 08:00 a.m. and 16:00 p.m. in the light phase. Only male rats were selected due to sex differences in behavioral memory tests. For example, male Wistar rats tend to navigate the platform more effectively than females in the MWM test (Morris water maze test) [18].

**Figure 1:**
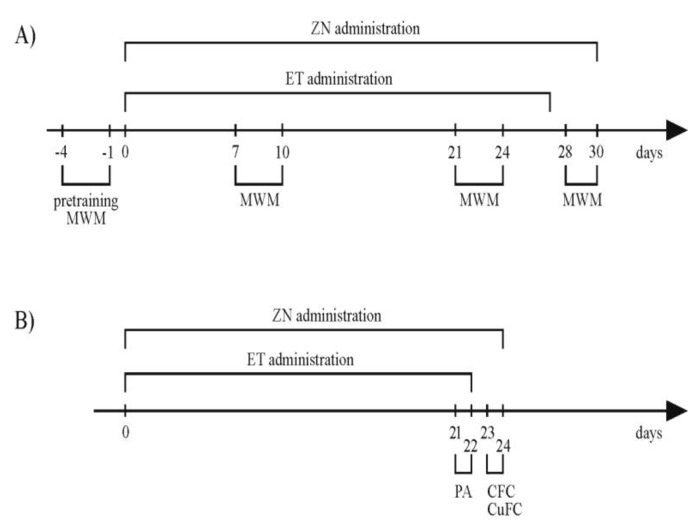
The timeline of the experiment procedures. (**A**) First phase and (**B**) second phase of the study. ZN-zonisamide; MWM-Morris water maze test; PA-passive avoidance test; CFC-contextual fear conditioning; CuFC-cued fear conditioning.

In the MWM test, the rats were divided into three groups: control (C), ethanol (ET), and experimental (ZN+ET) (Figure 1A). The doses of ethanol were based on a forced scheme of alcohol administration by Majchowicz [19] with Szmigielski’s amendment [20]. This ethanol intake model was used to eliminate the risk that observed differences would be induced by potential drug-induced low alcohol consumption. Ethanol was administered for three weeks in the ZN+ET and ET groups (respectively, n = 7, n = 8) (Figure 1A). The animals received 20% ethanol administered via an oral gavage at a daily dose of 5 g/kg (morning dose (08:00 a.m.) of 1.5 g/kg and afternoon dose (15:00 p.m.) of 3.5 g/kg). Additionally, the animals had access to 5% ethanol only between 16:00 p.m. and 08:00 a.m. In the other hours, the rats had access only to water. The control group (n = 7) received water and 1% methylcellulose solution.

In the PA and cued and contextual fear conditioning, the animals were divided into three groups, with seven rats in each group (control—C, ethanol—ET, and zonisamide +ethanol—ZN+ET) (Figure 1B). The scheme of alcohol administration was as described above.

The experiments were carried out in accordance with the Polish governmental regulations concerning experiments on animals (Dz.U.05.33.289), and the European Union Directive 2010/63/EU. All of the experimental protocols were approved by the Local Ethical Committee for Experimentation on Animals (resolution no. 77/ŁB/587/2011, 50/ŁB/754/2015).

### 2.2. Drugs

Zonisamide (Zonegran, Eisai^®^) was administered directly via an oral gavage to the stomach as a suspension in a 1% methylcellulose solution in the amount of 0.2 mL/100 g. The drug was administered repeatedly for three weeks at a dose of 50 mg/kg once a day only to animals from the experimental groups (ZN+ET (Figure 1A) and ZN+ET (Figure 1B)). During the behavioral trial days, the animals received zonisamide after the end of the test in order to avoid the results being influenced by the impact of the acute dose of the drug. The effect of zonisamide administered alone on memory processes was assessed in MWM and PA in our earlier study by Krupa-Burtnik et al. [21].

### 2.3. Morris Water Maze Test (MWM)

Spatial memory was assessed in the MWM test according to Williams et al. [22]. Undisturbed memory is described by a shorter tracking time and swimming distance, as well as longer time in the zone with a platform in the following days of the study. The test was performed in circular pool (180 cm in diameter; 50 cm high walls) with several extra-maze cues (water temp. 22–24 °C). The pool was placed in a quiet room and was virtually divided into four quadrants. A transparent circular platform (8 cm in diameter) was placed about 2 cm below the surface of the water in the center of selected section. During the experiment, the platform was invisible to the animals. The trials were recorded using ANY-maze 4.82 (ANY-maze, Wood Dale, IL, USA) with a camera hung above the pool, which allowed to monitor the test in real time without eye contact between the animal and the researcher.

The rats were pre-trained to learn the MWM scheme during the first three days of the experiment with the platform placed in a selected quadrant. In each trial, the animal was put into the pool in a different quadrant, facing the wall, and allowed to swim for 60 s. After finding the platform, the rat stayed on it for 15 s to observe the room. If the animal did not find the platform within the 60 s, it was placed there manually by the experimenter for 15 s to observe the room. Each animal underwent four trials per day with 60 s intervals between trials.

After three days of pre-training, the retention test without the hidden platform was performed in an analogous scheme. During these trials, the animals spent initially more time swimming in the quadrant that previously contained the platform; however, after failing to locate it, the rats then searched for it along the entire pool.

MWM was performed after the first and third week of ethanol administration and in the first week after the discontinuation of ethanol administration. Each week, the platform was moved to another quadrant and the activities of days 1–4 were repeated. During the alcohol administration period, the MWM trials were performed about three hours after the administration of a morning low dose of ethanol to prevent any possible effect of an acute dose of alcohol on animal behavior. Zonisamide was administered after the trials for the same reason.

### 2.4. Passive Avoidance (PA)

PA, which assessed the memory associated with fear, is a test based on the natural desire of a rodent to remain in dark places [23]. During the experiment, the rodents should learn to avoid selected compartments with an aversive stimulus. The step-through test was performed in the Gemini Avoidance System apparatus (San Diego, CA, USA) consisting of two compartments (25 × 20 × 17 cm) separated by a movable gate.

On the acquisition trial (first day of test), the rats went through a single trial. The test started with 60 s habituation in a dark compartment. Subsequently, the light was turned on and the gate was opened simultaneously, which should result in the animal moving to the second dark compartment. When the rat completely entered into second compartment, the gate automatically closed and a 3 s electric foot shock (0.5 mA) was delivered through the floor grid. Then, the animal was held in the dark compartment for approximately 15 s to associate an aversive stimulus with the environment. The apparatus was cleaned with 70% isopropyl alcohol after each trial.

The retention trial was performed 24 h after the acquisition trial. The rats were subjected to a similar trial lasting a maximum of 300 s. The foot shock was not delivered and the step-through latency to the dark compartment was measured. A longer step-through latency in the retention trial indicates an undisturbed memory of the aversive stimulus.

### 2.5. Contextual Fear Conditioning (CFC)

CFC is a basic behavioral test that assesses the memory of aversive stimulus and its association with the surroundings. After returning to a familiar environment, the rat demonstrates a freezing response as a memory of a former unpleasant experience. Freezing is defined as a lack of any activity, except for breathing. The higher percentage of freezing reflects an undisturbed memory of an aversive stimulus.

The study was performed in fear conditioning system (Ugo Basile, Gemonio, Italy), which consists of a sound-attenuating cage (55 × 60 × 57 cm) equipped with a light and speaker and an internal box with electrified floor (30 × 34 × 41.5 cm). Animal behavior was monitored and recorded using a camera located in the chamber.

The two-day study was performed according to the method described by [24]. The training session was performed on the first day and it began with 120 s adaptation with the new environment. In the next phase, the animal was exposed to 30 s sound signal (conditioned stimulus—CS) at a level of approximately 80 dB. A foot shock (unconditioned stimulus—US; 0.5 mA) was given to the animal during the last 2 s of the sound. Another analogous trial was performed after a 120 s break. At the end, the rat remained for about 1 min in the box to associate and consolidate information. The box was cleaned with 70% isopropyl alcohol during breaks to retain the same fragrance.

After 24 h, the animal was placed in the same box and the procedure was repeated under similar conditions (light, scent, and time of the test). The trial lasted 240 s, during which the total time of freezing responses was evaluated. The rats were not exposed to CS or US during the trial. The cage was cleaned with 70% isopropyl alcohol between trials.

### 2.6. Cued Fear Conditioning (CuFC)

CuFC is another test evaluating associative memory in rodents [24]. This behavioral model is similar to CFC and the only difference appears on the second day, when the animal is placed in the new environment and is exposed to a previously-known sound stimulus. Similar to CFC, foot shock is not used on the second day of the experiment.

This study involved the animals that had been used in the CFC, as the first day was similar to the CFC test. On the second day, the test was performed 3 h after completing the CFC trial. The appearance of the floor and walls had been changed and between trials the cage was cleaned with isopropyl alcohol with vanilla extract. After 180 s habituation, the rat was exposed to a familiar sound (80 dB) for 3 min, during which the total time of the freezing responses was evaluated.

### 2.7. Analysis of Results

The results are presented as median (horizontal bar), first and third quartiles (vertical column), and minimum and maximum (vertical line). Outliers and extreme values are represented with circles and asterisks. Non-parametric tests were chosen due to the non-normal distribution of the results. The normality of the distribution was checked by the Kolmogorov–Smirnov test, with Lilliefors correction. The results of the MWM were analyzed with the Kruskal–Wallis (ANOVA) test (comparison between groups) and Friedman rank sum test (comparison within a group). The data from the PA, CFC, and CuFC were analyzed using the Kruskal–Wallis (ANOVA) test, with treatment as a between-subject factor. A *p*-value of 0.05 or lower indicated a statistically significant difference for all of the statistical tests. The statistical analysis was performed using Statistica 13.1 (TIBCO Software, Palo Alto, CA, USA).

## 3. Results

The animals were exposed to a similar dose of ethanol. All of the animals from the ethanol (ET(A), ET(B)) and experimental (ZN+ET(A), ZN+ET(B)) groups received 20% ethanol via oral gavage twice a day. Moreover, the rats consumed a similar amount of alcohol during the night period. The animals from the experimental group (ZN+ET(A)) drank daily, on average, 22.48 ± 3.26 mL of ethanol and the rats from the ethanol group (ET(A)) drank, on average, 26.42 ± 5.14 mL of ethanol. The free-ethanol consumption rate was 3.32 ± 0.41 g/kg/day in the ZN+ET(A) and 3.41 ± 0.45 g/kg/day in the ET(A) group. In the ZN+ET(B) group from the fear-associated tests, the rats consumed a mean amount of 17.97 ± 4.42 mL of ethanol daily and those from the ET(B) group consumed 20.19± 1.33 mL of ethanol. The free-ethanol consumption rate was 2.79 ± 0.51 g/kg/day in the ZN+ET(B) group and 3.02 ± 0.27 g/kg/day in the ET(B) group. It is not possible to determine the effect of the drug on voluntary drinking alcohol due to the selected forced drinking model, which is undoubtedly a limitation of the study.

### 3.1. Pre-Training before Zonisamide Administration in MWM

All of the animals during pre-training had no memory impairment, which was indicated by the gradual decrease in time and traveled distance needed to find the platform. The time in the zone with the platform was also increased during the following days of the study (figures not included).

### 3.2. The Effect of One-Week Co-Administration of Zonisamide and Ethanol on the Spatial Memory in Rats in the MWM

**Figure 2:**
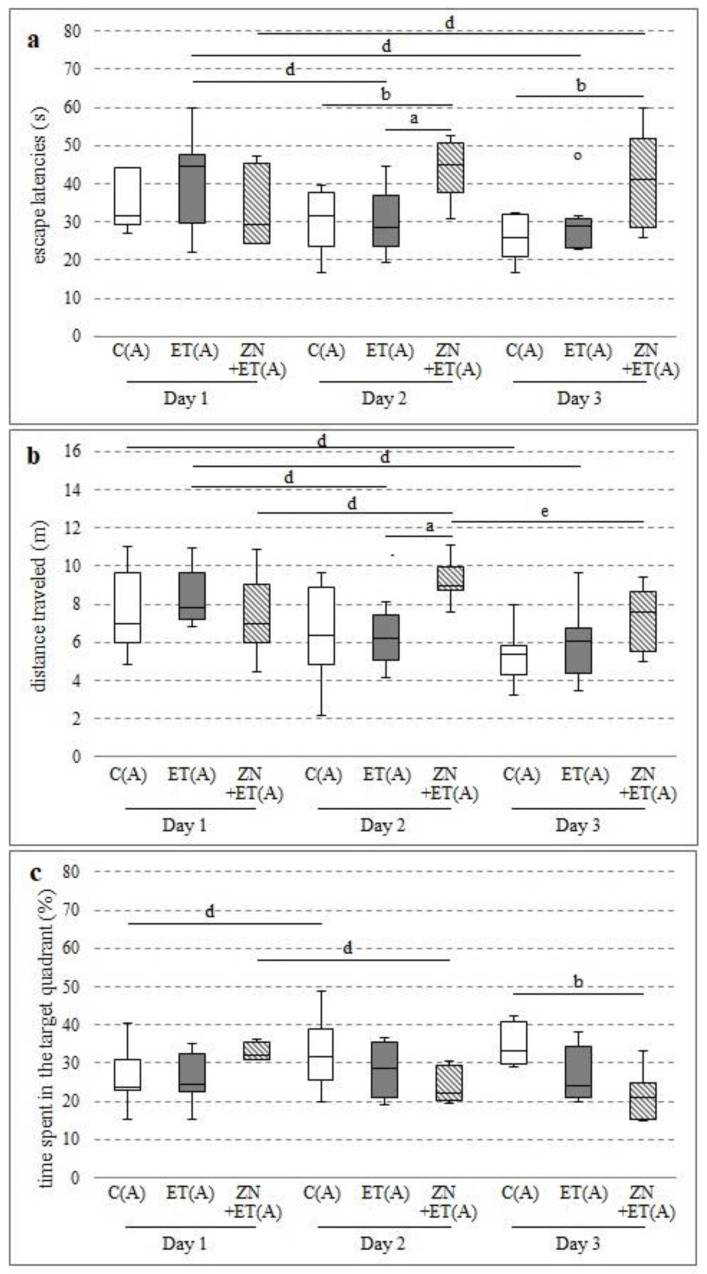
Effect of zonisamide in MWM after one week of alcohol administration on the time needed to localize the platform (**a**), the distance travelled by rats in order to localize the platform (**b**), the time spent in the zone with platform (**c**); C(A)—control group; ET(A)—ethanol group; ZN+ET(A)–zonisamide and ethanol group.^a^ Statistically significant difference between ET(A) and ZN+ET(A) on that day; *p* < 0.05, *Kruskal−Wallis test.* ^b^ Statistically significant difference between C(A) and ZN+ET(A) on that day; *p* < 0.05, *Kruskal−Wallis test.* ^d^ Statistically significant difference between the particular test day and test day 1; *p* < 0.05, *the Friedman’s test.* ^e^ Statistically significant difference between the particular 2 and 3 test days; *p* < 0.05, *the Friedman’s test.* The results are presented as median (horizontal bar), first and third quartiles (vertical column), and minimum and maximum (vertical line). Outliers are marked with circles.

Zonisamide given with ethanol significantly increased the time needed to find the platform after one week of administration. Statistically significant differences were observed on the second day, compared with the control and ethanol groups (H = 8.5798, N = 22, *p* = 0.0137), and on third day, compared with the control (H = 6.6014, N = 22, *p* = 0.0369) (Figure 2a). This time was also lengthened between days 1 and 3 in the ZN+ET(A) group (Chi sq = 7.1428, df = 2, *p* = 0.0281), but significantly shortened in the ET(A) group between days 1 and 2 (Chi sq = 6.75, df = 2, *p* = 0.0342) and between days 1 and 3 (Chi sq = 6.75, df = 2, *p* = 0.0342).

Zonisamide also extended the swimming distance in rats receiving alcohol on Day 2, and significant differences were observed compared with the ET(A) group (H = 9.1558, N = 22, *p* = 0.0103) (Figure 2b) and for the initial values (Chi sq = 10.5714, df = 2, *p* = 0.0051). However, these animals traveled significantly less distance on the next day (Chi sq = 10.5714, df = 2, *p* = 0.0051). Both the ET(A) and C(A) groups swam a shorter distance in the consecutive days and significant differences were observed between days 1 and 3 in both groups (Chi sq = 9.75, df = 2, *p* = 0.0076; Chi sq = 6, df = 2, *p* = 0.0498, respectively) and between days 1 and 2 in the ET(A) group (Chi sq = 9.75, df = 2, *p* = 0.0076).

On day 3, a significant decrease in time spent in the zone with the platform was observed in the animals from ZN+ET(A) compared with the C(A) group (H = 8.1395, N = 22, *p* = 0.0171) (Figure 2c). A significant decrease was also observed between days 1 and 2 in the ZN+ET(A) group (Chi sq = 8, df = 2, *p* = 0.0183). Time in the proper zone increased significantly between days 1 and 2 in the C(A) group (Chi sq = 8, df = 2, *p* = 0.0183), but in the ET(A) group it was comparable in the following days.

### 3.3. The Effect of Three-Week Co-Administration of Zonisamide and Ethanol on the Spatial Memory in Rats in the MWM

**Figure 3:**
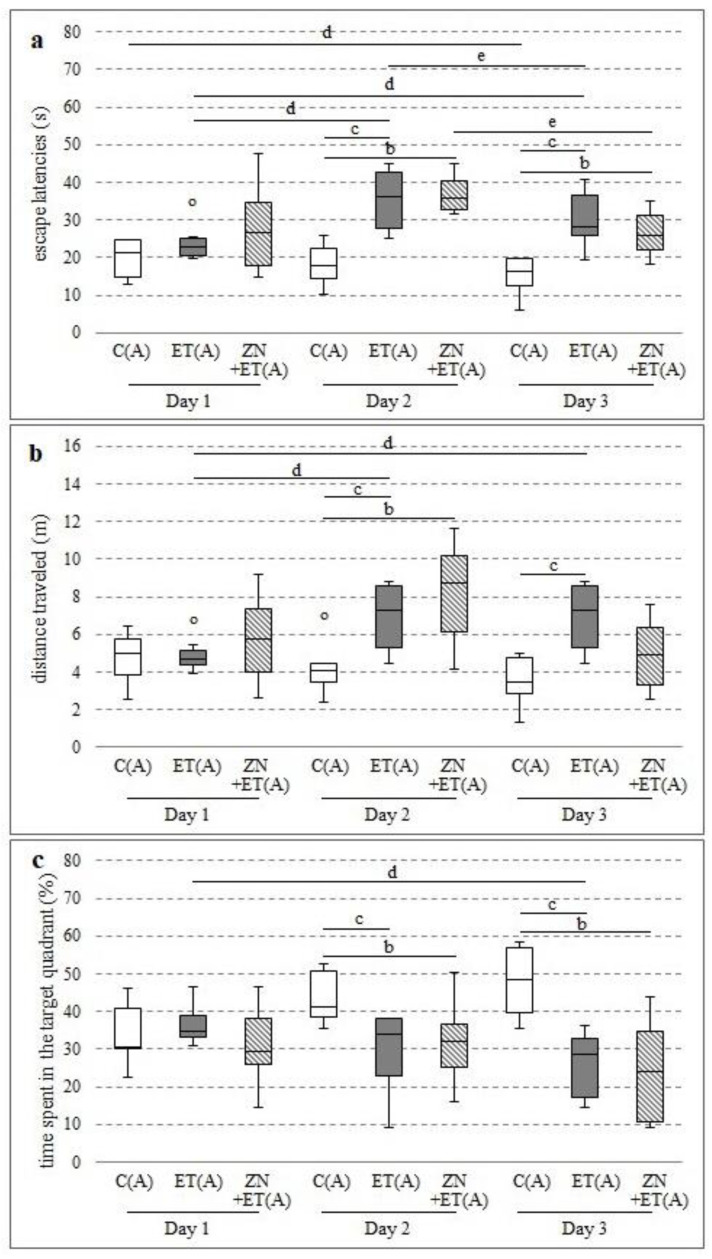
Effect of zonisamide in MWM after three weeks of alcohol administration on the time needed to localize the platform (**a**), the distance travelled by rats in order to localize the platform (**b**), the time spent in the zone with platform (**c**). ^b^ Statistically significant difference between C(A) and ZN+ET(A) on that day; *p* < 0.05, *Kruskal−Wallis test.* ^c^ Statistically significant difference between C(A) and ET(A) on that day; *p* < 0.05, *Krukal−Wallis test.* ^d^ Statistically significant difference between the particular test day and test day 1; *p* < 0.05, *the Friedman’s test.* ^e^ Statistically significant difference between the particular 2 and 3 test days; *p* < 0.05, *the Friedman’s test.* The results are presented as median (horizontal bar), first and third quartiles (vertical column) and minimum and maximum (vertical line). Outliers are marked with circles.

After three weeks, zonisamide co-administered with ethanol as well as ethanol prolonged the time needed to find the platform, and significant differences were observed on days 2 and 3 compared with the C(A) group (respectively H = 12.8041, N = 22, *p* = 0.0017; H = 12.1153, N = 22, *p* = 0.0023) (respectively H = 12.8041, N = 22, *p* = 0.0017; H = 12.1153, N = 22, *p* = 0.0023) (Figure 3a). Significant increases were also observed on days 2 and 3 in the ET(A) group compared with the initial values (Chi sq = 12.25, df = 2, *p* = 0.0022). However, this time significantly decreased between days 2 and 3 in the animals from the ZN+ET(A) and ET(A) groups (Chi sq = 7.7142, df = 2, *p* = 0.0211; Chi sq = 12.25, df = 2, *p* = 0.0022, respectively). The studied time also increased between test days 1 and 3 in the C(A) group (Chi sq = 7.714, df = 2, *p* = 0.0211).

Moreover, the animals from the ZN+ET(A) group traveled longer distances during the trial, and this increase was significantly different between the ZN+ET(A) and C(A) groups on Day 2 (H = 9.8588, N = 22, *p* = 0.0072) (Figure 3b). Ethanol also further lengthened this distance on days 2 and 3, compared with the C(A) group (H = 9.8588, N = 22, *p* = 0.0072; H = 8.3355, N = 22, *p* = 0.0155, respectively). In the ET(A) group, significant increases in distance were also noted between days 1 and 2, as well as between days 1 and 3 (Chi sq = 9.25, df = 2, *p* = 0.0098).

Furthermore, zonisamide co-administered with ethanol significantly decreased the time in the target zone on days 2 and 3 compared with the C(A) group (respectively H = 89,779, N = 22, *p* = 0.0112; H = 11.7404, N = 22, *p* = 0.0028) (Figure 3c). Similar decreases were observed on days 2 and 3 between the animals receiving ethanol and the control group (H = 8.9779, N = 22, *p* = 0.0112; H = 11.7404, N = 22, *p* = 0.0028, respectively). In the ET(A) group, the decrease on Day 3 was also significant compared with Day 1 (Chi sq = 6.25, df = 2, *p* = 0.04394).

### 3.4. The Effect of Zonisamide on the Spatial Memory in Rats in the MWM 12 h after Discontinuation of Ethanol Administration

**Figure 4:**
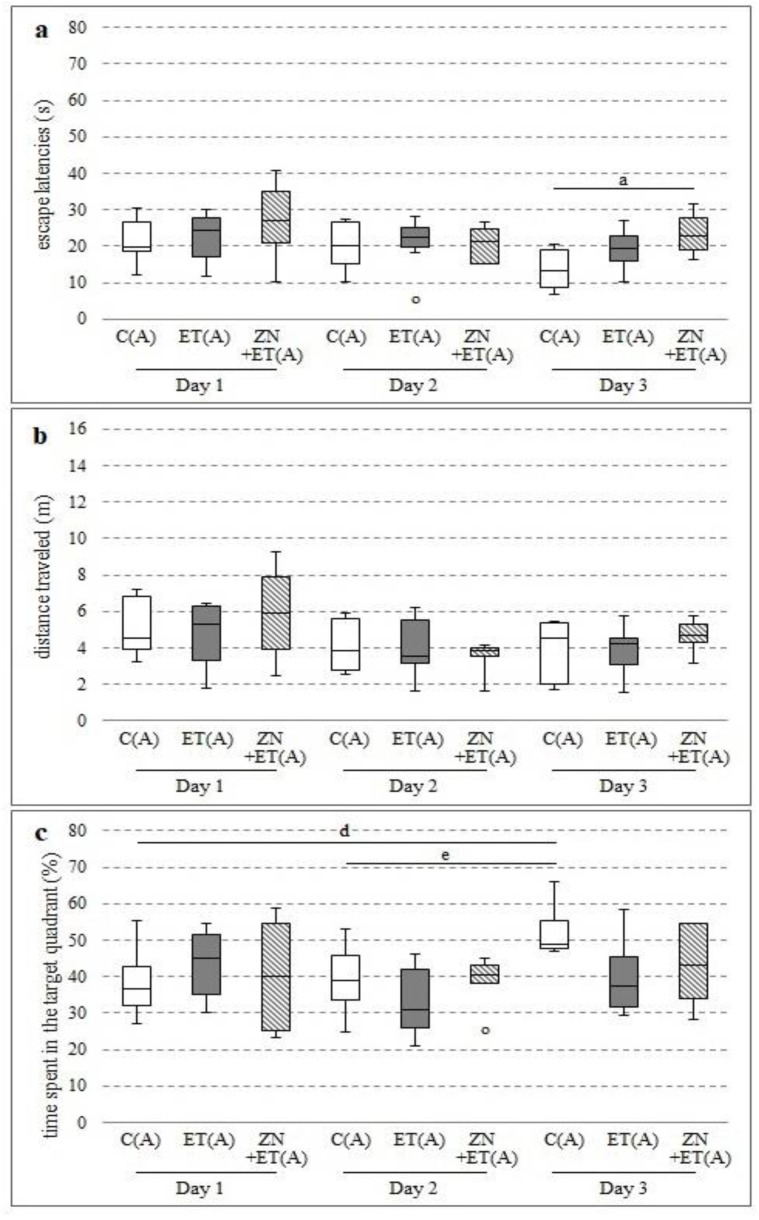
Effect of zonisamide in MWM after 12 h from the discontinuation of ethanol administration on the time needed to localize the platform (**a**), the distance travelled by rats in order to localize the platform (**b**), the time spent in the zone with platform (**c**). ^a^ Statistically significant difference between ET(A) and ZN+ET(A) on that day; *p* < 0.05, *Kruskal−Wallis test*. ^d^ Statistically significant difference between the particular test day and test day 1; *p* < 0.05, *the Friedman’s test*. ^e^ Statistically significant difference between the particular 2 and 3 test days; *p* < 0.05, *the Friedman’s test.* The results are presented as median (horizontal bar), first and third quartiles (vertical column) and minimum and maximum (vertical line). Outliers are marked with circles.

Compared with the C(A) group, zonisamide increased the time needed to find the platform on day 1 and 3 of the study, while a statistically significant difference was noted only on the last test day (H = 8.1441, N = 22, *p* = 0.017) (Figure 4a).

No differences between groups were observed for the traveled distance (Figure 4b).

In the C(A) group, significant increases in time spent in the target quadrant were noted only between days 1 and 3 and days 2 and 3 (Chi sq = 10.5714, df = 2, *p* = 0.0005) (Figure 4c).

### 3.5. The Effect of Zonisamide on the Memory in Rats in the PA

**Figure 5:**
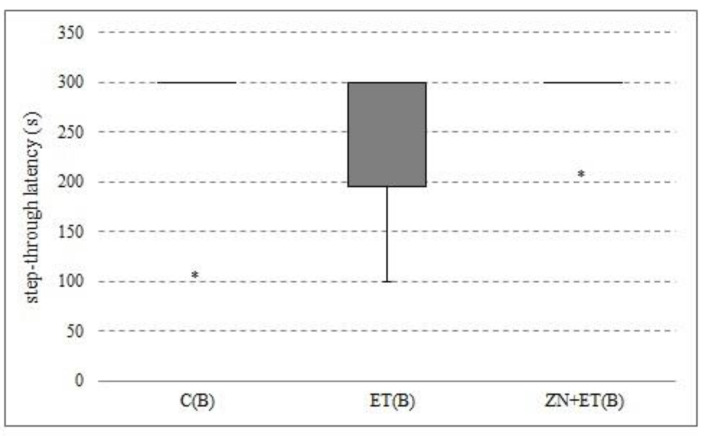
The effect of Zonisamide on memory in PA. The results are presented as median (horizontal bar), first and third quartiles (vertical column), and minimum and maximum (vertical line). Extreme values are marked with asterisks.

After three-weeks of administration of zonisamide and ethanol, no differences in step-through latency were found between groups (Figure 5).

### 3.6. The Effect of Zonisamide on Associative Memory in CFC 24 h after the Discontinuation of Ethanol Administration

**Figure 6:**
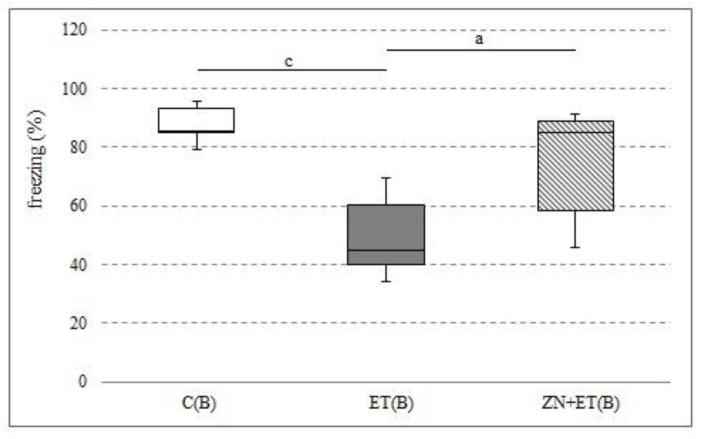
Freezing (%) after 24 h from the discontinuation of alcohol administration in the CFC. ^a^ Statistically significant difference between ET and ZN+ET on that day; *p* < 0.05, *Kruskal−Wallis test*. ^c^ Statistically significant difference between C and ET on that day; *p* < 0.05, *Krukal−Wallis test*.

Zonisamide administered at a dose of 50 mg/kg for three weeks significantly increased the proportion of freezing responses compared with the group receiving previously only ethanol (H = 11.5251, N = 21, *p* = 0.0031). This decrease in the ET(B) group was also significantly different to that in the C(B) group (H = 11.5251, N = 21, *p* = 0.0031) (Figure 6).

### 3.7. The Effect of Zonisamide on Associative Memory in CuFC 24 h after the Discontinuation of Ethanol Administration

**Figure 7:**
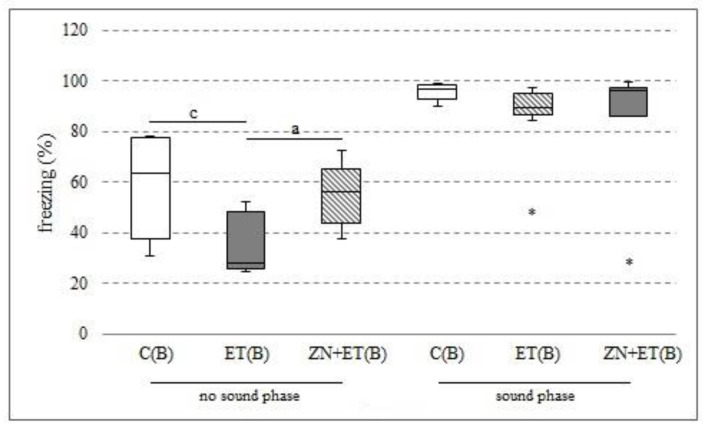
Freezing (%) after 24 h from the discontinuation of alcohol administration in the CuFC. ^a^ Statistically significant difference between ET(B) and ZN+ET(B) on that day; *p* < 0.05, *Kruskal−Wallis test*. ^c^ Statistically significant difference between C(B) and ET(B) on that day; *p* < 0.05, *Krukal−Wallis test.* The results are presented as median (horizontal bar), first and third quartiles (vertical column), and minimum and maximum (vertical line). Extreme values are marked with asterisks.

In the first 3 min habituation, the highest locomotor activity was demonstrated by the animals receiving previously only ethanol; they also demonstrated significant differences to the animals from the C(B) and ZN+ET(B) groups (H = 9.0241, N = 21, *p* = 0.011) (Figure 7). In the next 3 min of CuFC, during which the animals were exposed to the familiar sound, all of the animals demonstrated increased freezing behavior, and no significant differences were found between groups.

## 4. Discussion

The treatment of alcohol dependence is still a current research problem and medications such as disulfirame, acamprosate, naltrexone, and nalmefene are only modestly effective. New antiepileptic drugs such as topiramate have been investigated for their potential role in the treatment of alcohol dependence. The mechanism of their beneficial effect may be associated with their impact on various neurotransmission systems disturbed by ethanol [1,25]. Moreover, memory processes also play an important role in the development of addiction [15,16]. Zonisamide has a complex mechanism of action, which may offer a clinical advantage compared with current medicines [4,5,6,7]. The drug has a good long-term tolerability and the majority of adverse events seem to be mild-to-moderate [26,27]. Because of its multidirectional mechanism of action, the efficacy of zonisamide is investigated not only in the treatment of alcohol dependence [2,12,13], but also for other central disorders such as Parkinson’s disease and dementia with Lewy bodies [28], migraines [29], and eating disorders [30].

Our findings demonstrate that zonisamide has an adverse effect on memory processes during alcohol administration. In the MWM test, memory disturbances were observed in the zonisamide group after one week of alcohol exposure, which were reflected in a longer trial time and traveled distance than the control or ethanol group. Ethanol did not impair spatial memory, which was probably related to an insufficient period of alcohol administration. The limitations of the test should also be taken into account in the interpretation of the results. Some animals might have “freezing” reactions to shock and stress, which disturb the assessment of time and distance. However, such behavior was observed only once in our study and did not affect the overall results. After three weeks of the experiment, memory impairment persisted in drug-treated animals and in rats receiving ethanol alone. These results are consisted with those of previous clinical trials, indicating that zonisamide may cause a modest decrease in verbal fluency and working memory [2]. Moreover, simultaneously with this study, we assessed the impact of the drug administered alone on memory in MWM, but these results were published earlier. It was found that the prolonged administration of zonisamide is associated with memory disturbance, but the effect seemed to be transient as changes occurred only on a single day of the experiment [21]. In contrast, zonisamide appeared to have a beneficial effect on spatial memory in a gerbil model of global forebrain ischemia. Pre- and post-ischemic drug administration at a dose of 150 mg/kg *i.p.* improved spatial memory in MWM, and a neuroprotective effect related to a reduction in pyramidal cell damage in the hippocampal CA1 region was noted in animals pre-treated with zonisamide [31].

In the present study, the assessment of spatial memory in MWM was continued following the discontinuation of alcohol administration. It is also known that spatial memory disturbance may occur in people with alcohol use disorder undergoing alcohol detoxification [32]. After 12 h from the last dose of ethanol, some memory disturbances were observed, reflected in an increase in the trial time in rats receiving zonisamide on the third day of the experiment. However, the observed disturbance occurred only once and concerned only one studied parameter.

Topiramate, an antiepileptic drug with a similar mechanism of action to zonisamide, was also investigated for its effects on spatial memory in MWM in rats receiving ethanol. Three-week administration (60 mg/kg p.o.) reduced alcohol-induced memory impairment, and beneficial effects were also observed during the period of abstinence, especially in the second week [33].

The next research model used in this study was the PA test. No effects on memory were observed in animals repeatedly receiving zonisamide together with ethanol or ethanol alone. In our previous study, it was observed that zonisamide disturbed memory after 7 or 14 days of administration at a dose of 50 mg/kg [21]. Moreover, ethanol administration by a modified liquid diet at an increased concentration impaired memory processes in the rats, reflected in a decrease in passive avoidance latency. The same negative effect was observed in rats during ethanol withdrawal [34].

In the CFC test, the expected response of the animal is to remain frozen, which is described as a lack of any activity other than breathing. This non-activity demonstrates the correct association of an unpleasant stimulus with the environment by the animal. The reaction indicates proper functioning of memory processes and constitutes an anxiety response [35]. The results of this study show that ethanol impaired memory associated with fear after 24 h from the last dose, and zonisamide improved responses, which indicates that the drug has a potential beneficial effect on anxiety-related memory processes. These results are consistent with those of previous preclinical trials indicating that ethanol pretreatment impaired conditioned freezing to context cues in adult rats [36]. In another study, chronic intermittent ethanol exposure increased anxiety-like behaviors in rats, which was related to increased synaptic excitability in the ventral hippocampus [37].

Similar to the contextual test, the CuFC test assessed the time that the animal remained motionless. In the first stage of the experiment, in which the animals were acquainted with the new environment, the animals previously given ethanol demonstrated an increased motor activity. However, this ethanol-induced activity level was decreased by zonisamide. In the second stage of the study, the memory of the aversive stimulus was undisturbed in all of the studied groups.

The effect of certain antiepileptic drugs, including zonisamide, on the level of anxiety was also tested in an open-field test. Based on its natural preference, rats feel safe in dark and closed places, and exposure to an open space is a stressful factor. The authors did not observe any significant differences in locomotion or anxiety after 12 weeks of administration of zonisamide [38]. The results from the clinical trial also showed that Zonisamide administered during alcohol detoxification reduces anxiety, assessed with the Hamilton Anxiety Rating Scale, and this effect was more pronounced in the zonisamide group than in participants treated with diazepam [14]. Moreover, zonisamide might be effective as an adjunctive therapy of refractory anxiety in patients [39].

## 5. Conclusions

In summary, the effect of zonisamide on memory processes in animals receiving ethanol is varied and depends inter alia on the type of memory. The disruptive effect of the drug on spatial memory in the MWM test deserves emphasizing. Nevertheless, our present results suggest also that the drug may have some potential beneficial effect after the discontinuation of ethanol administration, which was associated especially with associative memory in the CFC test. The obtained results indicate the necessity for further studies in order to fully understand the mechanism of this interaction and its influence on learning processes.

## Data Availability

The data presented in this study are available on reasonable request from the corresponding author. The data are not publicly available due to privacy.
